# Study of the effects of bio-silica nanoparticle additives on the performance, combustion, and emission characteristics of biodiesel produced from waste fat

**DOI:** 10.1038/s41598-023-46140-w

**Published:** 2023-11-02

**Authors:** Ravikumar Jayabal, Gopinath Soundararajan, R. Ashok Kumar, Gautam Choubey, Yuvarajan Devarajan, T. Raja, Nandagopal Kaliappan

**Affiliations:** 1 Department of Mechanical Engineering, Saveetha School of Engineering, SIMATS, Saveetha University, Chennai, Tamil Nadu India; 2 Department of Mechanical Engineering, KCG College of Technology, Chennai, Tamil Nadu India; 3 Department of Mechanical Engineering, RMD. Engineering College, Chennai, Tamil Nadu India; 4 Department of Mechanical and Aerospace Engineering, Institute of Infrastructure Technology Research and Management (IITRAM), Ahmedabad, Gujarat 380026 India; 5 Material Science Lab, Saveetha Dental College and Hospitals, SIMATS, Saveetha University, Chennai, Tamilnadu India; 6 Department of Mechanical Engineering, Haramaya Institute of Technology, Haramaya University, Dire Dawa, Ethiopia

**Keywords:** Environmental sciences, Engineering, Mechanical engineering

## Abstract

Numerous countries are investigating alternative fuel sources in response to the escalating issue of energy inadequacy. Using environmentally sustainable biodiesel as a potential alternative to fossil fuels, particularly from waste sources, is a developing prospect. This study aims to examine the feasibility of utilizing industry leather waste as a diesel fuel substitute. Traditional transesterification was used to obtain methyl ester out of leather waste. After processing, 81.93% of methyl ester was produced. Bio-silica (Bio-Si) is used as a fuel additive to enhance combustion and decrease emissions. This work utilized a leather industry waste fat biodiesel (LIWFB), LIWFB blend (B50), LIWFB blend with Bio-Si nanoparticles (B50Bio-Si50, B50Bio-Si75, and B50Bio-Si100 ppm) to analyze the engine outcome parameters at standard operating conditions. Experimental results revealed that adding Bio-Si in the biodiesel blend increased thermal brake efficiency (BTE) but was lower in diesel fuel. The biodiesel blends reduced NOx emissions more than Bio-Si nanoparticle blends. Furthermore, the smoke opacity was reduced by 31.87%, hydrocarbon (HC) emissions were reduced by 34.14%, carbon monoxide (CO) emissions were decreased by 43.97%, and oxides of nitrogen (NOx) emissions were slightly increased by 4.45% for B50Bio-Si100 blend compared to neat diesel. This investigation determined that all the emissions remained lower for all combinations than neat diesel, with a small increase in NOx emissions. Therefore, the LIWFB blend with Bio-Si nanoparticles was a viable diesel fuel alternative in diesel engines.

## Introduction

The expansion of transportation infrastructure, the rapid growth of road transportation, and the demand for massive transportation can all contribute to the increase in fuel consumption. Diesel engines have lower fuel consumption, higher efficiency, and exceptional economy and effectiveness. In contrast, it is observed that diesel engines exhibit higher levels of smoke opacity and NOx emissions. There is a trade-off between these two emissions^1,2^. Emissions from diesel engines are hazardous to both human and environmental health^3–5^. Alternatives to conventional diesel fuel are being researched all over the globe, considering numerous factors such as affordability, technical adequacy, economic aspects, environmental satisfaction, etc.^6–8^. Biodiesel can be derived from vegetable oil, animal fat, and cooking oil. The increasing rate of environmental degradation and a significant increase in fossil fuel consumption demonstrate the viability of biodiesel as a potential alternative to diesel fuel^9,10^.

Biodiesel, a renewable fuel, may be used in diesel engines. Transesterifications are environmentally friendly; long-term feedstocks produce biodiesel without aromatics or sulfur^11–13^. A growing country like India has ample capacity to produce biodiesel from animal fat, waste seed, waste peel, and other waste sources, all of which improve the performance of diesel engines^14–16^. The fleshing waste from the leather industry would be among that bioenergy. Around 15 million tonnes of hides and skins can be processed into leather annually worldwide. The tanning process generates over 6 million tonnes of solid waste annually. One of the most significant environmental contamination problems is the massive amount of sludge, or around 4.5 million tonnes, disposed of from effluent treatment plants^17^. Biodiesel extracted from leather industry waste fat, the fat acid value was 0.28 mg KOH/g. Therefore, the transesterification method was used to turn the fleshing fat into biodiesel. To determine the ideal transesterification conditions necessary to create biodiesel, they employed methanol and NaOH^18–21^. Waste leather biodiesel produced lower cylinder pressure and a higher heat release rate (HRR) than neat diesel, assessed to B20 and B30 blends, and the B10 blend displayed 4% and 6% greater BTE. The biodiesel blends increased NOx emissions.

Further, B10 reduced smoke, HC, and CO emissions^22^. The leather waste methyl ester (LWME) has been tested on a diesel engine. Results revealed that the BTE was lower for the biodiesel mixture, and the BSEC was greater because they are inversely proportional. Compared to diesel, LWME30D70 had NOx emissions that were 9.84% higher, while CO emissions, HC emissions, and smoke opacity were reduced by 22.70%, 48%, and 6.43%^23^.

Plenty of research has demonstrated that using fuel additives can effectively enhance the efficiency of diesel engines and mitigate emissions. Utilizing fuels mixed with nanoparticles attracts significant interest due to their notable efficiency in reducing pollutants and improving combustion. Increasing the nanoparticle’s maximum energy and surface area facilitates an augmented contact surface between the fuel and oxidant, enhancing the catalytic activity^24–26^. More investigations revealed that adding feasible nanoparticles with biodiesel improves engine and fuel allocations. Less ignition delay, increased density of energy, combustions, flaming enthalpy, and lower exhaust emissions were noted when utilizing nanoparticles as catalysts (fuel-born)^27^. Copper oxide nanoparticles were mixed in the blend, which reduced fuel consumption and raised BTE^28^. In adding Al_2_O_3_ and CeO_2_ nanoparticles with test blends in the diesel engine, the BTE increased, and exhaust emissions were decreased^29^. Biodiesel and ferrofluid nanoparticles are fueled in diesel engines, and results found that adding 1% nanoparticles increases 17% BTE, and 1.5% addition decreases BTE^30^. Saravankumar et al.^31^ used biodiesel blend doping with silicon oxide nanoparticles in diesel engines, and they found that NOx and CO_2_ emissions were raised. CO emissions and HC emissions were reduced.

Several studies have shown that metal-based nanoparticle compositions improve the quality of fuel used while reducing exhaust pollutants^32–34^. Bio-Si nanopowder can successfully be produced from cogon grass. To ensure complete burning, the dried cogon grass leaves were placed outside. As a result of the burning process, ash was formed. After sun-drying, the cogon grass ash was crushed to a fine powder with a mill crusher. After cooling for 10 h, fine ash was collected from the grinding machine. The fine cogon grass ash was then used to make the Bio-Si.

Additionally, they may be produced in nano size because of technical development. They can store internal energy and possess unique thermal behavior^28^. The engine’s overall performance improved dramatically when Bio-Si was combined with biodiesel, reducing the engine’s hazardous emissions^27^.

## Novelty and motivation of the research

The primary aim of this study was to produce biodiesel using fat derived from industrial leather waste, with the intention of potentially replacing diesel fuel and thereby mitigating engine exhaust emissions, as supported by existing research. More research needs to be conducted on the LIWFB in diesel engines. Moreover, there needs to be more studies regarding utilizing LIWFB + Bio-Si nanoparticle blends in a CRDI diesel engine. The primary aims of this study were to investigate the use of a mixture of LIWFB and Bio-Si nanoparticles as a fuel additive in a diesel engine and to evaluate its performance compared to that of pure diesel fuel.

## Materials and methods

### Raw oil extraction

**Figure 1:**
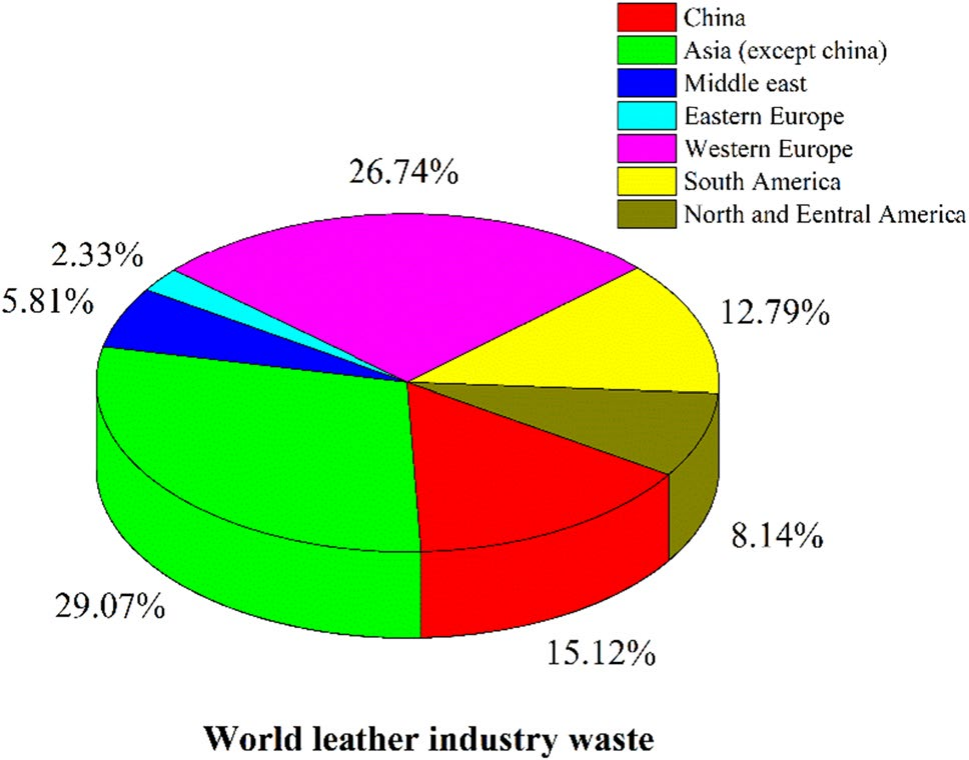
Word leather industry waste.

In Vellore, Tamil Nadu, India, leftover fat from the leather industry was gathered. The world leather industry waste is given in Fig. 1. Examples of fatty leather waste feedstock obtained from industrial effluent waste include degreasing, wetback, and chamois wash water. These materials comprise HC-rich fatty oil that can be incinerated to generate pure oil for biodiesel conversion. Raw oil water content and other impurities were removed, and the feedstocks were obtained in varying quantities and warmed at over 100 °C. Although wetback comprises greater fat than other leather wastes, the amount varies. The study’s maximum yield, which was used to make fat oil, was achieved using wetback and chamois wash water, each 250 ml under ideal operating circumstances, according to Table 1. After the water in the flask reaches boiling point, some water escapes as steam, while the remainder condenses at the bottom. During heating, the raw fat oil accumulated on the top portion of the container. Raw oil was removed using the syphon method at room heat and kept in a bottle to produce biodiesel.

**Table 1:** Raw oil extraction from LIWF.

Degreasing (ml)	Wetback (ml)	Chamois (ml)	Temperature (°C)	Warming time (min)	Yield of oil (ml)
200	100	200	> 100	40–45	55–60
100	200	200	> 100	40–45	75–80
200	100	200	> 100	40–45	55–60
–	200	200	> 100	70–95	180–190
50	200	250	> 100	60–75	170–180
–	250	250	> 100	80–90	230–240

## Transesterification process

**Figure 2:**
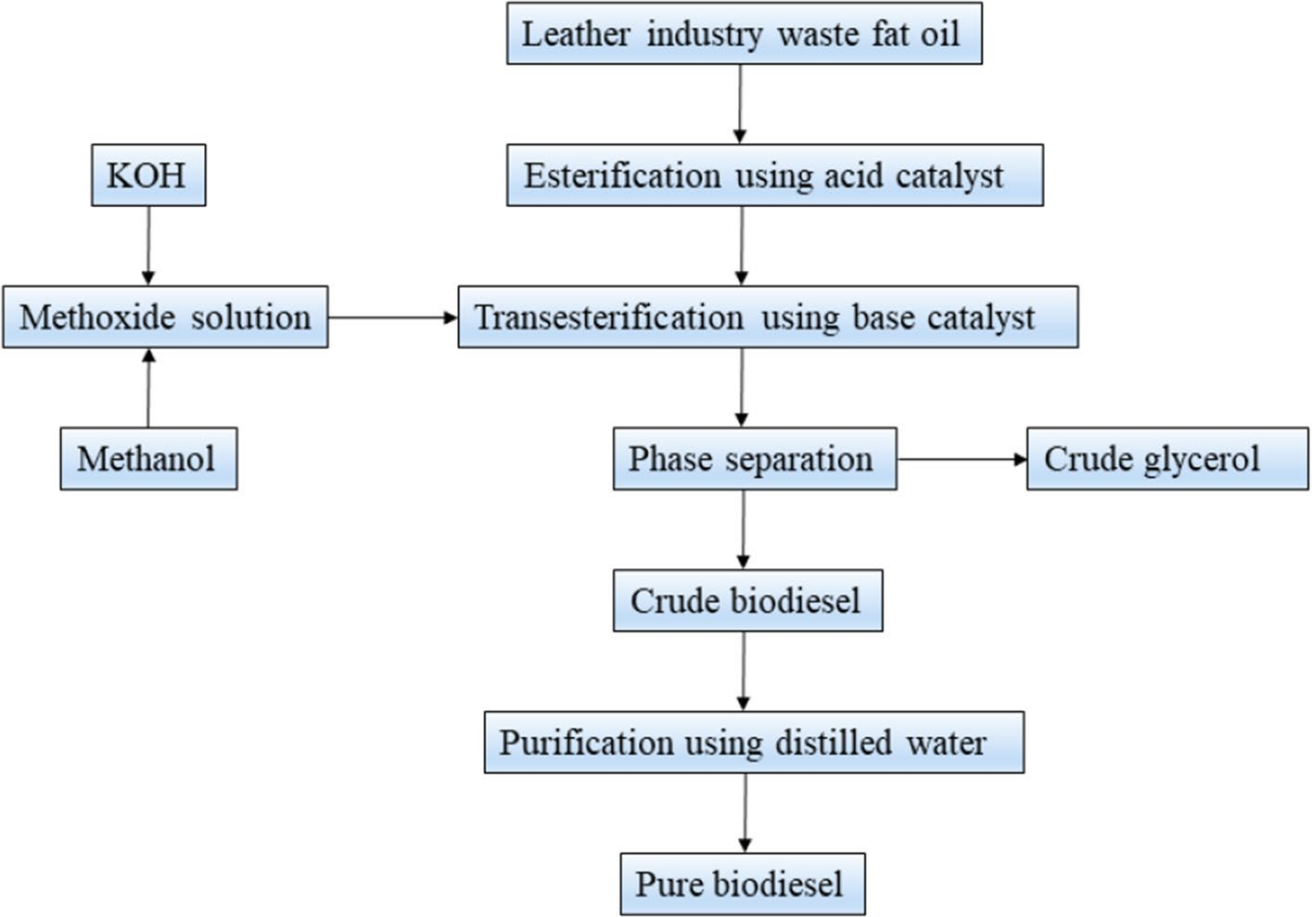
Biodiesel production process.

In this experiment, biodiesel was produced by the transesterification method. Raw fat oil is extracted and heated to 55 °C. The standard working conditions for the transesterification process include a temperature of 60 °C, a reaction period of 90 min, a catalyst concentration of 1% potassium hydroxide, and an agitation speed of 600 rpm. The ratio between methanol and oil in this method is 8:1. The experimental study encompassed the modification of methanol to oil ratios, which were altered within the range of 6:1–12:1. The experimental conditions involved maintaining the reaction temperature within the range of 70–80 °C while varying the catalyst concentration from 0.5 to 2%. It is possible to cool the esterified products to 40 °C in a settling tank, with the esterified oil remaining at the reactor’s base. At the same time, the methanol–water combination rises to the top. It is sent to the purification column, where the methanol can be separated from the concoction and reused. The wedge column combines the transesterification product with the top layer of methanol and biodiesel before separating the mixture into the purification column. The recovered methanol is used well, while the remaining biodiesel undergoes a series of washes to remove impurities. The biodiesel production process is shown in Fig. 2. The biodiesel physiochemical properties are given in Table 2.

**Table 2:** Physiochemical properties of biodiesel.

Properties	LIWFB
Kinematic viscosity, CST @ 40 °C	6.12
Density (kg/m^3^)	884
LHV (MJ/kg)	38.2
CCI	42
Flashpoint °C	165
Fire point °C	173
Cloud point °C	4
pour point °C	− 3
Oxidation stability %	10.4
Water content mg/kg	428.03
Acid value mg KOH/g	0.34

## Bio-Silica nanoparticle preparation

**Figure 3:**
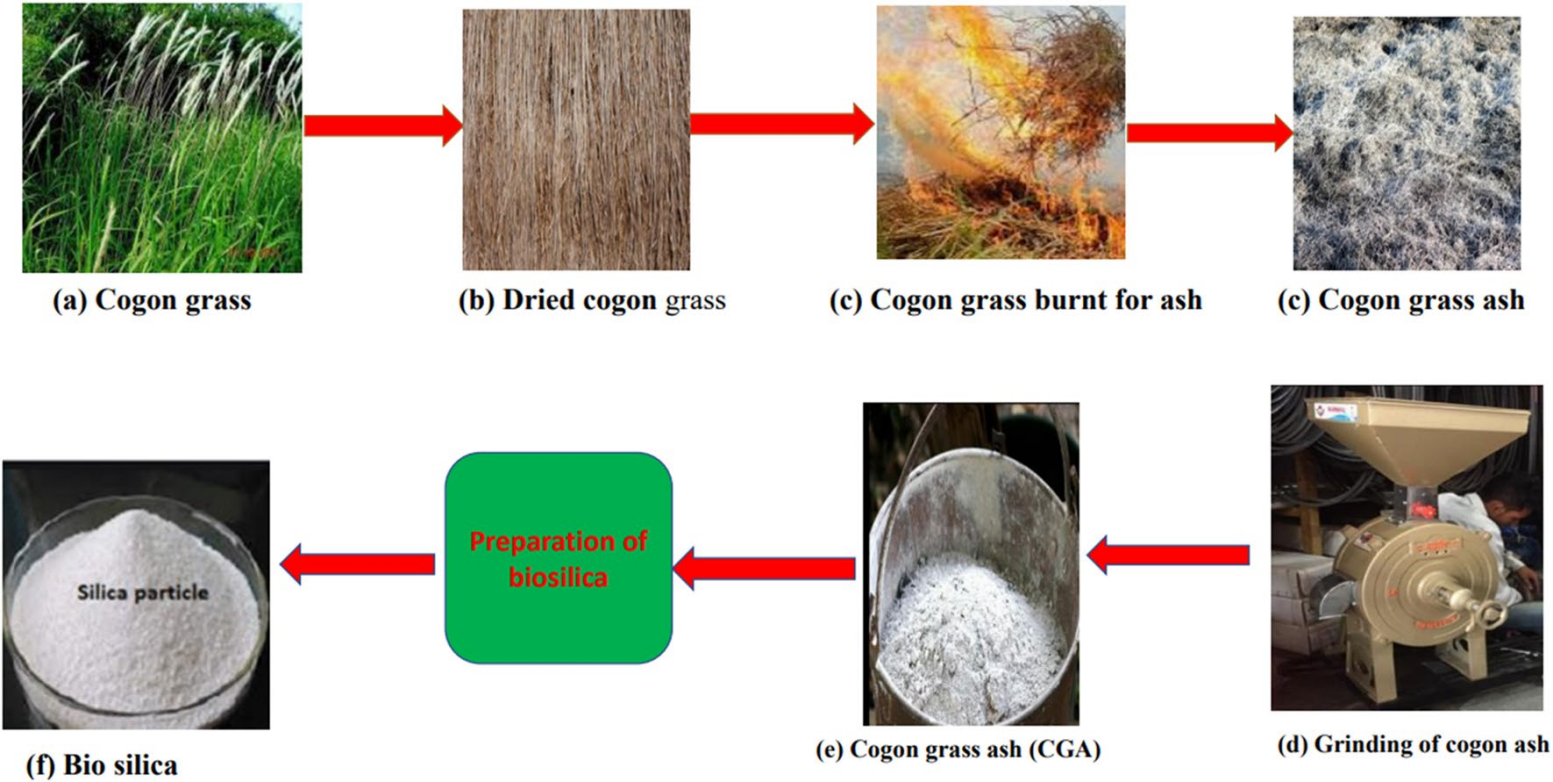
Preparation of bio silica from cogon grass.

Cogon grass was collected from Nallamala Forest, Andhra Pradesh, and was utilized for preparing the Bio-Si, as shown in Fig. 3a. Large quantities of Bermuda grass are available in the village region in India. Figure 3b shows the dried cogon grass for 48 h in sunlight. The dried cogon grass leaves were placed outside to ensure total combustion, as seen in Fig. 3c. Ash was produced because of the burning process. The cogon grass ash was processed into a fine powder using a mill crusher after sun-drying, as illustrated in Fig. 3d. Fine ash was removed from the grinding machine after cooling for 10 h, as shown in Fig. 3e. The fine cogon gras ash was further used for preparing the Bio-Si shown in Fig. 3f.

## Test fuel preparation

Bio-Si nanoparticles are mixed with LIWFB blend to analyze the properties of the baseline and customized fuels. An ultrasonicator was employed for an hour to ensure the fuels were mixed correctly. The blending procedure is additionally optimized using the mechanical stirrer. The testing fuels are LIWFB (B100), LIWFB-diesel blend (B50), and diesel-LIWFB-Bio-Si blends (B50Bio-Si50, B50Bio-Si75, and B50Bio-Si100 ppm). The testing fuels have been operating consistently for the last 30 days to evaluate the stability of biodiesel with diesel. In the subsequent investigation, there was no way to distinguish biodiesel from diesel, and the mixtures were robust enough even to justify the additional investigation.

## Experimentation unit and facilities

**Figure 4:**
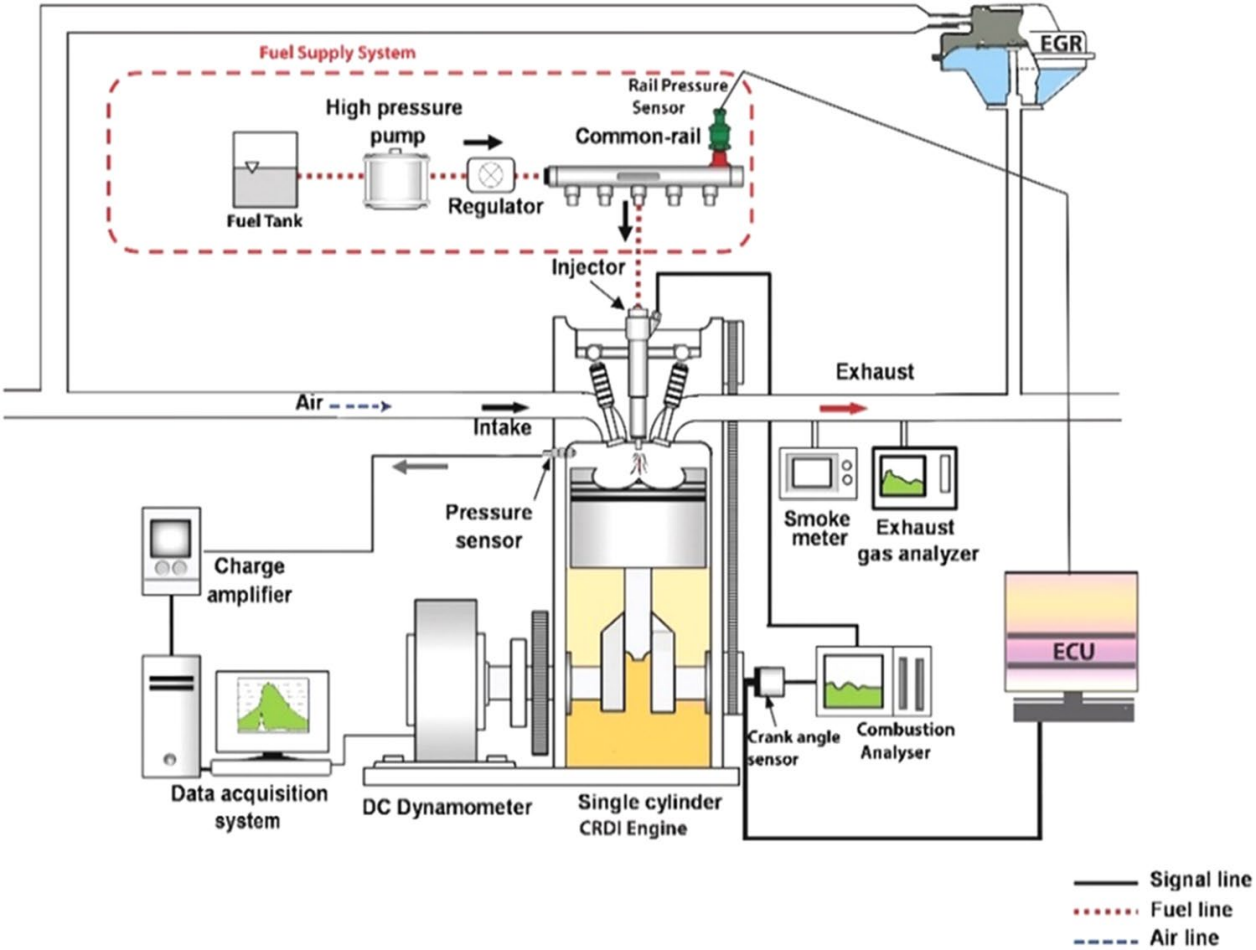
Experimentation setup.

Figure 4 depicts the experimentation unit configuration. The test was done in TV1, Kirloskar diesel engine. The test engine configuration consisted of a water-cooled method linked to a dynamometer and a 1-cylinder, 4-stroke, VCR engine. An open electronic control unit (ECU), sensors, and transducers were added to the engine to meet the specifications. The AVL 437C smoke meter and the AVL 444N digas 444N analyzer were used to measure NOx and smoke emissions, respectively.

A common rail direct injection (CRDI) structure was necessary to accomplish the appropriate fuel injection pressures for the experiment. Fuel injection was modified to meet the engine CRDI system, and a high-pressure pump was fitted to the filter. It is connected to the common fuel supply line to store the fuel and maintain constant fuel supply pressure. The pressure sensor is coupled to the Nira i7r ECU and fitted to a common fuel line to keep fuel injection pressure. A six-hole solenoid-controlled fuel injector has been selected to attain the required fuel injection pressure. The prototype sensors and actuators were modified using the ECU to ensure every component functioned completely. If the engine runs properly, it is deemed ready for evaluation. The test engine description is given in Table 3.

**Table 3:** Descriptions of the test engine.

Particulars	Descriptions
Make and model	Kirloskar, TV-1
Type	One-cylinder, 4-stroke, water-cooled
Bore and stroke	87.5 mm and 110 mm
Cubic capacity	661 cc
Compression ratio	17.5
Engine speed	1550 rpm
Injection pressure	600 bar
Injection timing	23° CA bTDC
Rated power output	3.5 kW at 1500 rpm

## Test procedure

Initially, baseline data were gathered by operating an engine fueled with neat diesel. The steady-state condition engine was run for 5 min before each observation to ensure accuracy. The five brake power factors (0, 1.29, 2.58, 3.87, and 5.16 kW) of the engine were tested, along with 1500 rpm speed, 600 bar injection pressure, 23° CA bTDC injection timing, and a compression ratio of 17.5; all these factors were used in the engine trials. The influence of a LIWFB mixture with Bio-Si nanoparticle addition (50, 75, and 100 ppm) on engine parameters was examined in the test engine. On observations of the cylinder pressure, the combustion characteristics of the study were developed. The influence of cyclical changes is minimized by the HRR computation by pressure measurements over 100 cycles’ mean value. The lubricating oil was maintained at a temperature of 85–90 °C. A 15-min continuous run of the test engine allowed for recording. The dependability values have been aggregated over the three test iterations.

## Error analysis

The limitations included in any investigation include factors such as measuring preciseness, appropriate calibration, and atmospheric conditions, which contribute to the overall reliability of the results. In this work, a comprehensive uncertainty analysis has been conducted to identify and quantify those errors accurately. Uncertainty assessment provides a comprehensive clarification of the repetition of scientific research. The overall uncertainty assessment of the engine outputs is determined by employing the root mean square approach. The AVL Digas 444 N emissions analyzer was employed to quantify the emissions of test fuels. Total sampling uncertainty (TSU) is estimated for each engine operation test circumstances utilizing Eq. (1), where $\Delta \mathrm{U}$ is total uncertainty, ${\Delta \mathrm{x}}_{1}, {\Delta \mathrm{x}}_{2}, {\Delta \mathrm{x}}_{3}\dots {\Delta \mathrm{x}}_{\mathrm{n}}$ are the errors of ${x}_{1}$, ${x}_{2}, {x}_{3}\dots {x}_{n}$. Table 4 lists the range, resolution, and accuracy of the devices.

**Table 4:** Range, resolution, and accuracy of devices.

Quantity measured	Range	Resolution	Accuracy
Speed	400…6000 min^−1^	1 min^−1^	$\pm$ 1 min^−1^
Oil temperature	0–125 °C	1 °C	$\pm$ 4 °C
Air flow rate	0–500	1%	$\pm$ 1 mm
Exhaust gas temperature	0–1000 °C	0.1%	$\pm$ 2 °C
Pressure measurement	0–110 bar	0.1%	$\pm$ 0.1 bar
Carbon monoxide	0–15%	0.01% volume	$\pm$ 0.02%
Carbon dioxide	0–20%	0.01% volume	$\pm$ 0.3%
Hydrocarbon	0–30,000 ppm	1 ppm volume	$\pm$ 10 ppm
Smoke	0–100%	0.1%	$\pm$ 1%
Oxides of nitrogen	0–5000 ppm	1 ppm volume	$\pm$ 5 ppm

1 $$\Delta U=\sqrt{{\left(\frac{\partial U}{{\partial x}_{1}}{\Delta x}_{1}\right)}^{2}+{\left(\frac{\partial U}{{\partial x}_{2}}{\Delta x}_{2}\right)}^{2}+{\left(\frac{\partial U}{{\partial x}_{3}}{\Delta x}_{3}\right)}^{2}+\dots {+\left(\frac{\partial U}{{\partial x}_{n}}{\Delta x}_{n}\right)}^{2}}$$

## Results and discussions

### Leather industry waste fat (LIWF) oil characterization

**Figure 5:**
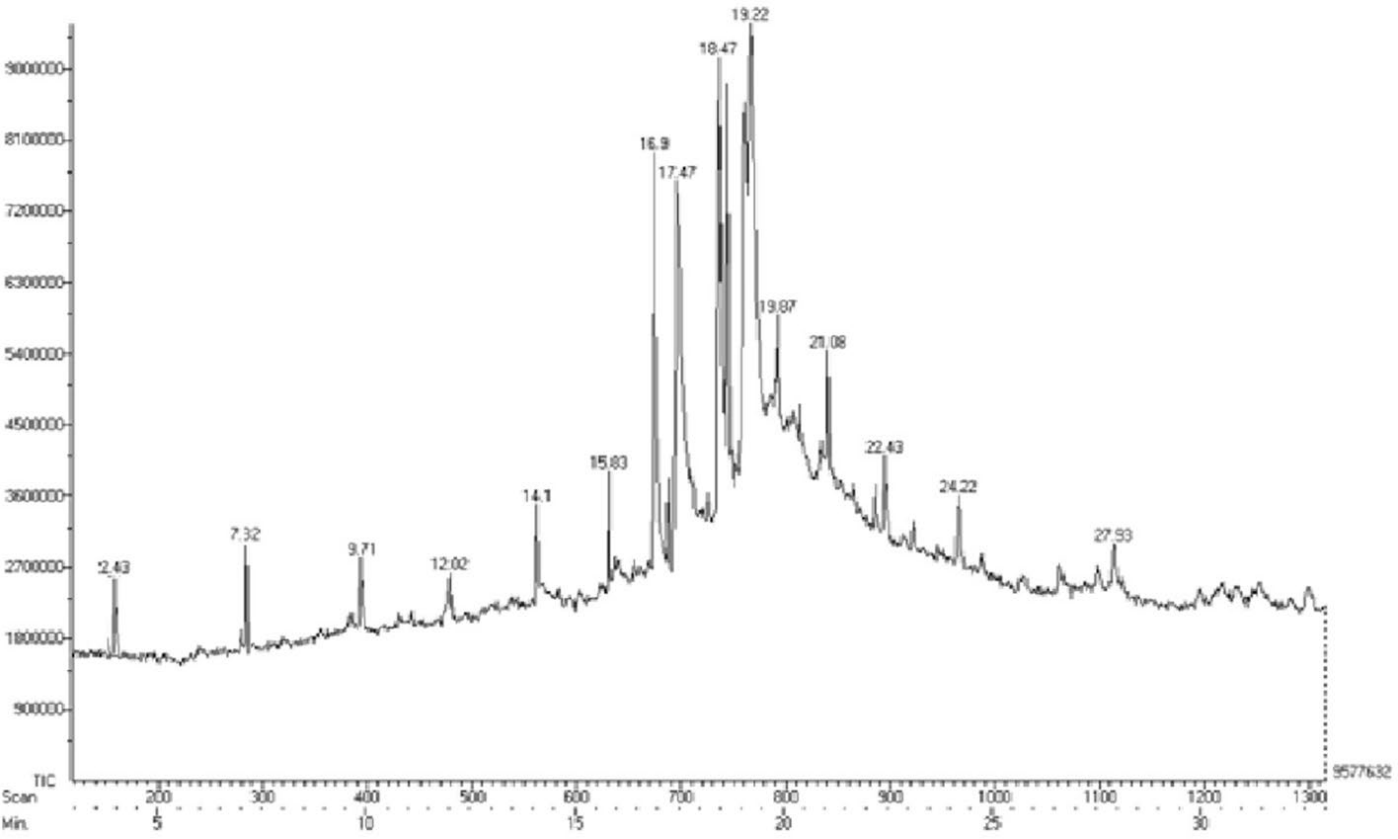
GC–MS analysis of LIWF oil.

Gas chromatography-mass spectrometry (GC–MS) equipment was used to analyze the LIWF oil at the Sophisticated Analytical Instrument Facility (SAIF) of the Indian Institute of Technology, Madras. The GC system employed was an AGILANT 7890 equipped with a Flame Ionisation Detector (FID) and a headspace injector. The MS instrument used was a JEOL AccuTOF GCV, capable of detecting mass within the range of 10–2000 atomic mass units (amu) with a mass resolution of 6000. The GC–MS analysis is a valuable technique employed to discern and characterize various chemical compounds and their respective compositions inside pyrolytic oil. A volume of 1 μl of the sample oil was introduced through injection, and afterward, the obtained spectra were compared to the NIST mass spectral database library. The findings of the GC–MS analysis of LIWF oil are illustrated in Fig. 5.

## Bio-Silica nanoparticle preparation and characterization

**Figure 6:**
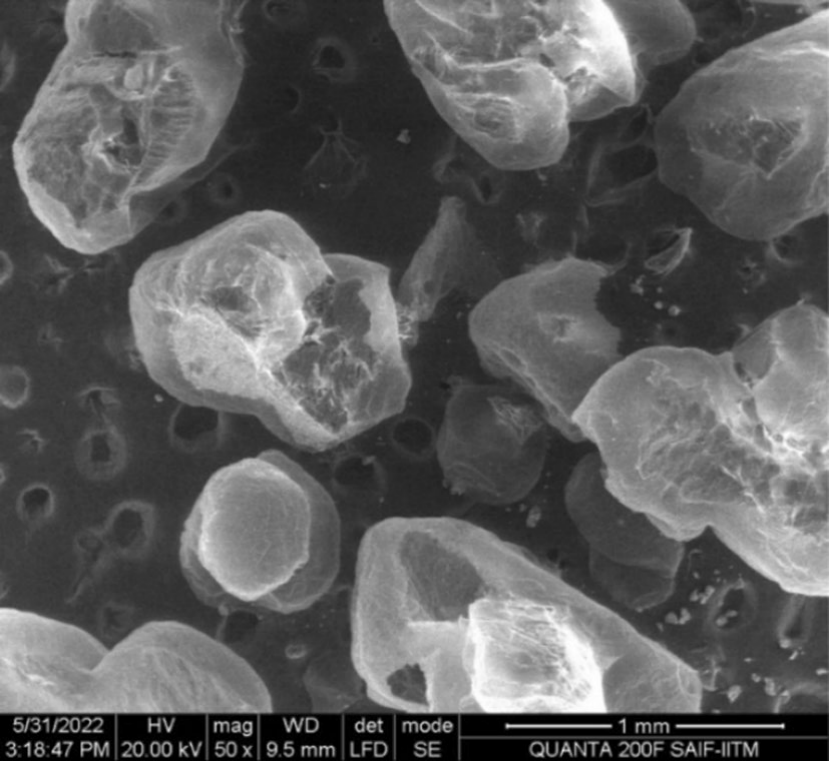
SEM of Bio-Si nanoparticles.

**Figure 7:**
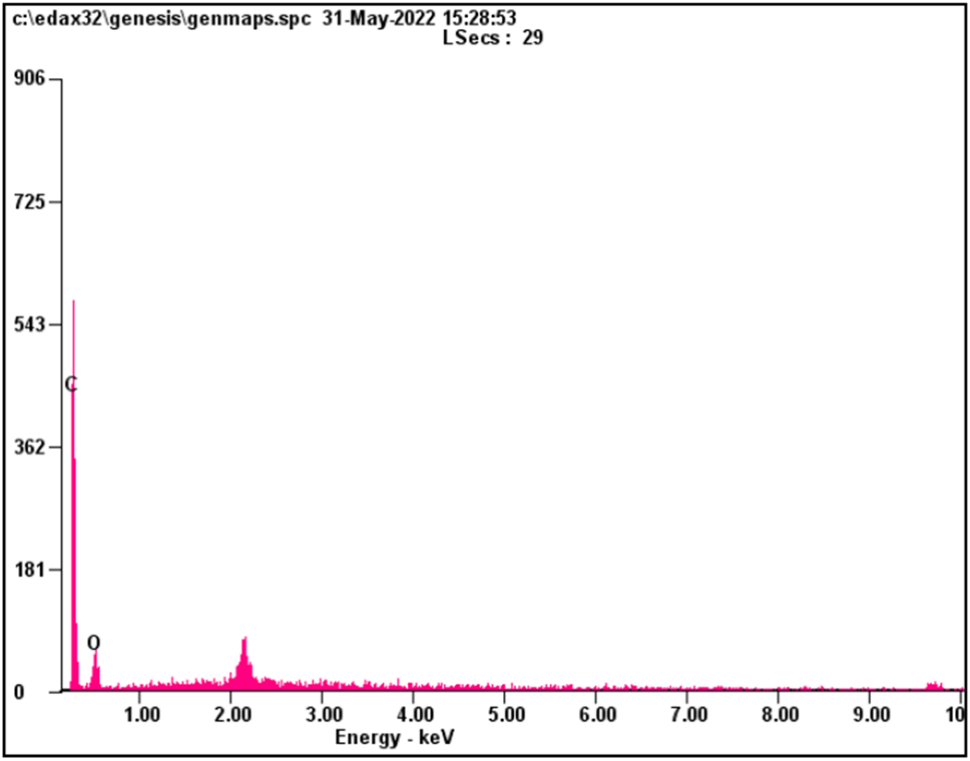
EDX spectrum of Bio-Si nanoparticles.

Scanning electron microscopy (SEM) was utilized to examine the morphology of Bio-Si, and the discovered Bio-Si nanoparticle’s spherical shape was verified. Figure 6 shows an SEM image of Bio-Si. The findings of an energy-dispersive X-ray (EDX) analysis of Bio-Si nanoparticles are displayed in Fig. 3. High-purity carbon nanoparticles (79.72% weight and 83.96% atomic) and oxygen nanoparticles (20.28% weight and 16.04 atomic) are both visible as peaks in the spectrum of the compound (Fig. 7). The average size of bio-Si is 10–20 m. The nanoparticles are scattered in the solution using a stirrer. The characteristic of the nanoparticles is a more extensive surface area and tremendous surface energy. The mixed nanoparticles create micro molecules, which start to settle. The nanoparticles must have their surface altered or adjusted to stabilize them in a fluid. A cationic surfactant called cetyl trimethyl ammonium bromide (CTAB) harms the nanoparticle surface by coating its surface to prevent particle deposition. The magnetic stirrer method was employed to diffuse nanoparticles in the bottom. Weighed Bio-Si (50–100 ppm) and CTAB were added to an ethanol solvent. Then, the mixture was magnetically stirred for roughly two hours until it became a nanofluid^27^. Table 5 lists the physical characteristics of the test fuel.

**Table 5:** Test fuel specifications.

Properties	Density (at 15 °C) (kg/m^3^)	Kinematic viscosity (40 °C) (mm^2^/sec)	Calorific value (MJ/kg)	Calculated cetane index
Standard	ASTM D1298	ASTM D445	ASTM D240	ASTM D976
Diesel	840	3.3	42.5	47
LIWFB	884	6.12	38.2	42
B50	862	4.71	40.35	44.5
B50Bio-Si50	828.5	3.43	39.93	39.25
B50Bio-Si75	824.5	3.32	40.03	41.5
B50Bio-Si100	822.5	3.23	40.26	42.8

### Combustion characteristics

The combustion process can be obtained from pressure data in an internal combustion engine. The pressure within an engine cylinder during combustion can provide insight into the efficiency of fuel combustion and the amount of power generated. This analysis uses pressure data to study combustion by measuring the pressure within a cylinder using a pressure transducer. To determine ignition timing, peak pressure, and pressure rise rate, this data can be analyzed. The heat release rate, which describes how much heat is released during combustion, is one parameter that can be calculated from pressure data. The heat released during combustion can be calculated using the first law of thermodynamics, which relates the change in the system’s internal energy to the heat released.

A pressure smoothing technique removes noise from pressure data measured in an internal combustion engine. Sensor errors, mechanical vibrations, and other sources of measurement uncertainty can cause noise. To smooth pressure data, the first step is to collect raw data from the pressure transducer. To capture the full details of the pressure signal, the data is collected at a high sampling rate. Raw pressure data is then filtered to remove any high-frequency noise. Low-pass filters are commonly used to smooth pressure, removing frequencies above a certain threshold. A baseline drift or offset may affect the pressure signal in some cases. A change in the surrounding environment’s temperature or pressure can cause this. The pressure signal can be corrected for this by applying a baseline correction. In the next step, data is smoothed after filtering and baseline corrected. Smoothing removes any remaining noise and makes the pressure signal more uniform. Moving average, Savitzky-Golay and wavelet smoothing are a few of the smoothing algorithms that can be employed. The moving average algorithm is used in this study. Moving average algorithms are commonly used to smooth pressure data from internal combustion engines. The algorithm replaces the centre point of the window with the calculated average of a window of adjacent data points. A smoothed signal is produced by shifting the window one point at a time and repeating the process.

Moving averages can be represented mathematically as follows in Eq. (2),

2 $${y}_{i}= \frac{1}{N} x \,sum(x\left(i-j\right), j=0\, to \,N-1$$

where N is the window size, which is the number of adjacent data points used to calculate the average value; y_i_ is the smoothed output value at time step i, x(i−j) is the input data value at a time step j steps before i, and x(i−j) is the input data value at a time step j steps prior to i. Using this equation, the algorithm calculates the average value of the window of size N centered on data point i. As a result of averaging the input values x(i−j) within the window, y(i) represents the output value at time step i.

Data on pressure can be used to calculate several parameters, such as combustion efficiency, which indicates how efficiently fuel is burned, and combustion duration, which indicates how long it takes for combustion to take place. HRR measures the energy released by an internal combustion engine as a function of time. An engine’s performance and emissions are greatly affected by this parameter. Wiebe functions are commonly used to calculate the HRR from pressure data.

As a function of crank angle, the Wiebe function describes the heat release rate. This can be expressed as follows in Eq. (3),

3 $$Q= {Q}_{m} x \left\{1-\mathrm{exp}\left[-\beta x {\left[\frac{\theta }{{\theta }_{0}}\right]}^{n}\right]\right\}$$

Q is the heat release rate as a function of crank angle (J/deg), Q_m_ is the maximum heat release rate (J/deg), β, n, and θ_o_ are parameters that determine the shape of the function, and θ is the crank angle in degrees. The Wiebe function parameters can be calculated based on pressure data by determining the point of inflection or first and second derivatives of the pressure curve. Equation (4) can be used to calculate the heat release rate.

4 $$Q= -V x \frac{dp}{d\theta } x \left[\frac{1}{\gamma -1} \right]x \left[\frac{1}{{\eta }_{com}}\right] x\left[ \frac{360}{2\pi }\right]$$

The volume of the cylinder (m^3^), the pressure concerning the crank angle, and the ratio of specific heat and combustion efficiency are used to calculate HRR.

#### Cylinder pressure analysis

**Figure 8:**
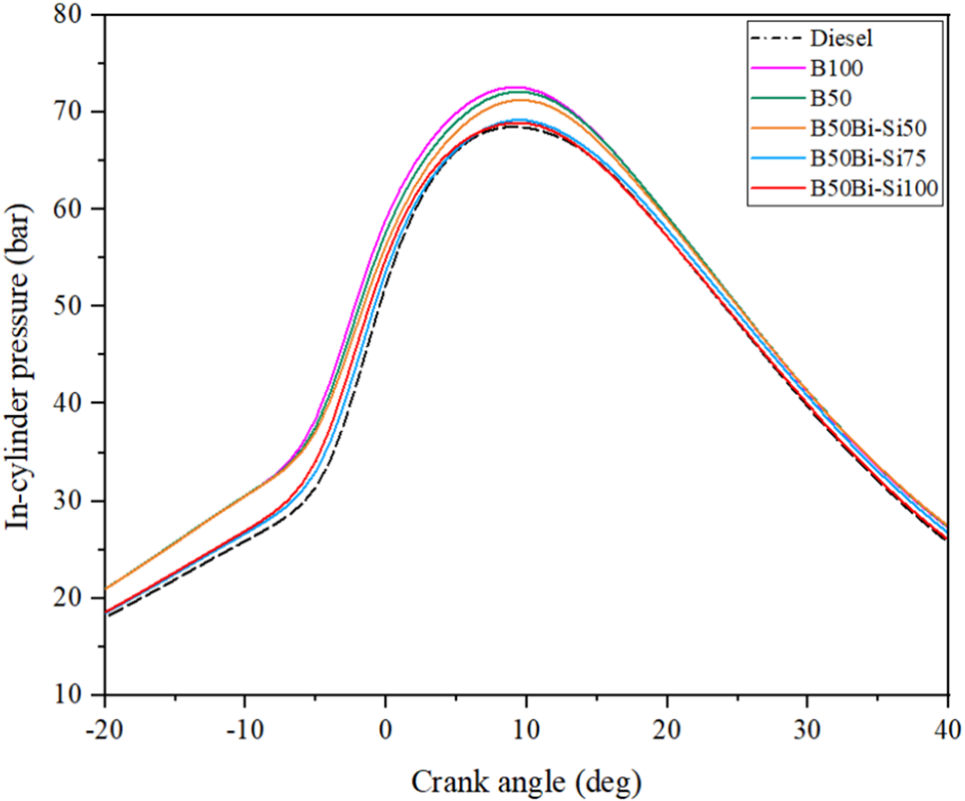
Variations of in-cylinder pressure.

A diesel engine’s combustion level is controlled by adjusting the amount of fuel injected into the premixed combustion area, which affects the maximum pressure produced. Figure 8 illustrates the variants in the pressure within the combustion chamber (CC). The peak in-cylinder pressure in B100 is 5.80% higher than in diesel.

This can be ascribed to the elevated oxygen concentration in biodiesel, which facilitates the ignition process and consequently increases peak pressure^35^. Adding Bio-Si as a dopant in the biodiesel blend indicates a steady enhanced cylinder pressure than diesel due to the nanoparticles’ enhanced contact surface area and the elevated oxygen concentration in biodiesel. The combustion process within the cylinder is accelerated due to the fuel mixture’s composition and air’s presence^36,37^.

#### Heat release rate analysis

**Figure 9:**
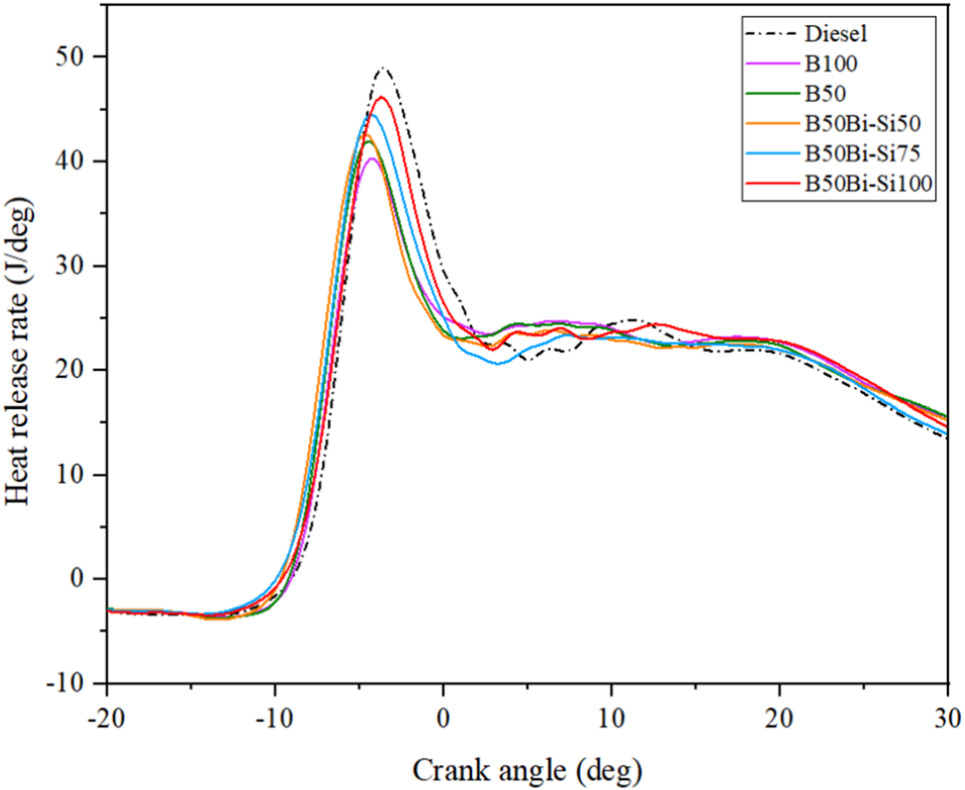
Variations of HRR.

The HRR enhances the chemical energy discharged from the fuel through combustion. The HRR is determined using the in-cylinder pressure measurements. Adding Bio-Si to the blend ensures complete fuel combustion and prevents carbon deposits on the CC walls^38^. Figure 9 demonstrates the lower HRR in LIWFB and its mixture related to neat diesel owing to the low calorific values potential to produce less heat^2^.

The blend B50Bio-Si100 produces higher HRR related to other blends but is 5.1% less than diesel. It implies that the rise in HRR, which is unique in biofuel and diesel, is solely due to the emergence of Bio-Si. Before the fuel enters the CC, reactions raise their HRR. Since Bio-Si is present, effective premix step atomization reduces the delay time and enhances combustion^36,38^.

### Performance characteristics

#### Brake thermal efficiency

**Figure 10:**
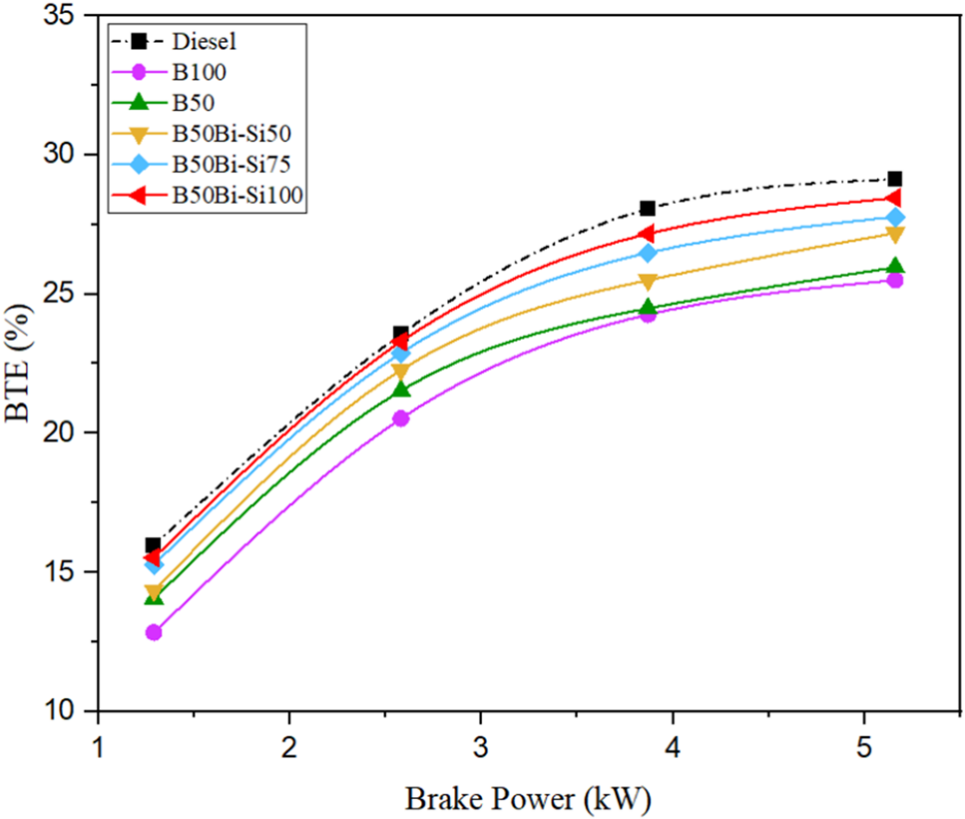
Variations of BTE.

An engine BTE is a valid measure for calculating the quantity of fuel power transferred into work. According to the data portrayed in Fig. 10, it can be perceived that the BTE exhibited an upward trend when the braking power increased throughout all test mixes. Increasing brake power delivers extra fuel to the ignition chamber, resulting in more noticeable ignites, generating extra heat, and improving BTE^39^. Figure 8 indicates that the test fuels had decreased BTE contrasted to diesel owing to the lower heating value, necessitating extra fuel to push into the CC and provide an equivalent engine output power^40^. Among the test fuel, the blend B50Bio-Si100 produces 11.60% higher BTE compared to B100 at maximum load owing to the nanoparticle’s high-level flame temperature, extended flame sustenance, physical delay period of ignition, and higher evaporation rate can improve BTE, but 2.27% lower than diesel fuel^36^.

#### Brake-specific energy consumption

**Figure 11:**
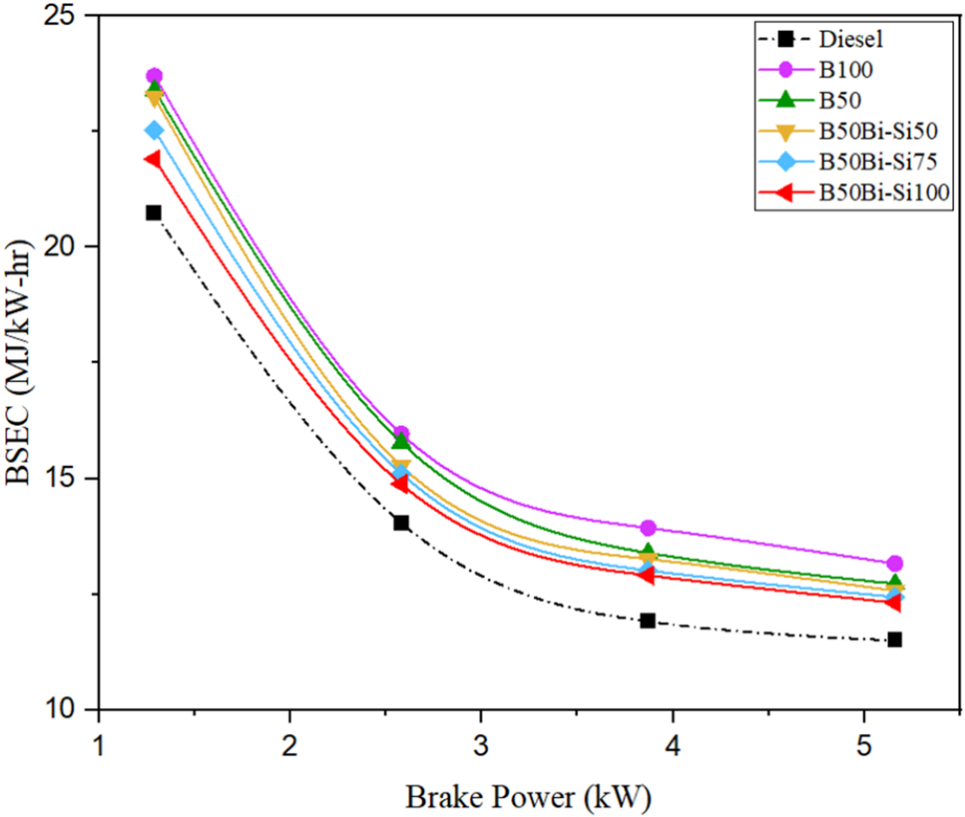
Variations of BSEC.

The quantity of energy created inside a CC while burning a specific fuel is described as brake-specific energy consumption (BSEC). Fuel calorific content and brake-specific fuel consumption combine to produce BSEC^41^. Since BTE and BSFC have an opposite relation, fuel with a lower BTE has a higher BSFC. Hence, the rationale behind the variations in BTE observed in biodiesel, biodiesel-Bio-Si blends, and diesel may similarly be extended to BSFC^42^. Figure 11 shows BSEC reducing while raising brake power. The BSEC for all test fuels exhibited an increase compared to diesel fuel. This can be attributed to the fact that the fuel blends possessed lower heating values, hence requiring a greater amount of fuel to achieve a similar plunger movement in the injection pump^35^. The mixture B50Bio-Si100 generated lesser BSEC compared to other blends owing to the higher additive of Bio-Si but higher than diesel. To achieve excellent ignition quality and good atomization of mixed fuels with Bio-Si nanoparticles, the BSEC is enhanced^37^. Because of the positive effects of Bio-Si, it has plenty of oxygen to complete the combustion process. There will be a decrease in BSFC due to the fuel’s physical characteristics^43^.

### Emissions analysis

#### Oxides of nitrogen

**Figure 12:**
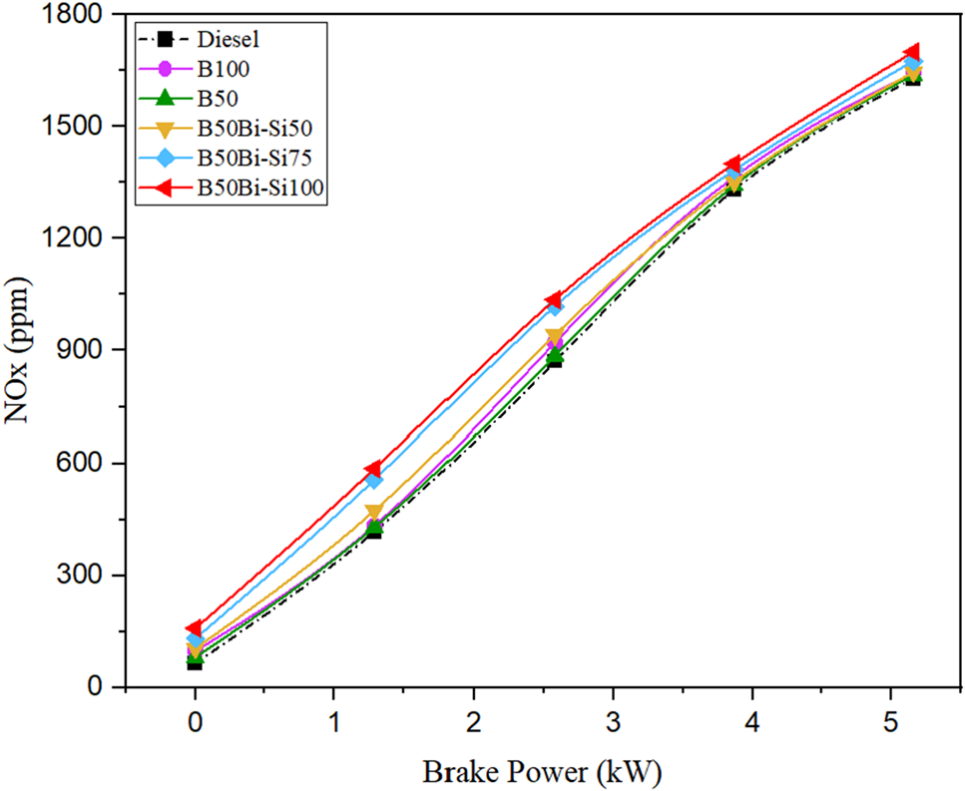
Variations of NOx emission.

The primary factors influencing NOx emissions include the temperature of the burned mixture, the air–fuel ratio at the local level, and the duration of the reaction. Figure 12 illustrates the changes observed in NOx emissions among the tested fuels. According to Abbaszaadeh et al.^44^, fuel-born oxygen in blended mixes leads to a higher combustion rate, resulting in increased NOx emissions than diesel fuel. The observed reduction in NOx emissions could be attributed to two factors: the diminished volatility of biodiesel, leading to a slower evaporation process compared to diesel fuel, and the absence of aromatic compounds^45^. The B50Bio-Si100 produces 4.35% higher NOx emissions than diesel fuel at maximum brake power. According to the Zeldovich reaction mechanism, adding Bio-Si in biodiesel increases NOx emissions by improving fuel combustion inside the engine cylinder, resulting in high temperatures. As a result, NOx emissions levels during combustion are higher than conventional fuel. The HRR and cylinder pressure are higher^36,37^.

#### Smoke opacity

**Figure 13:**
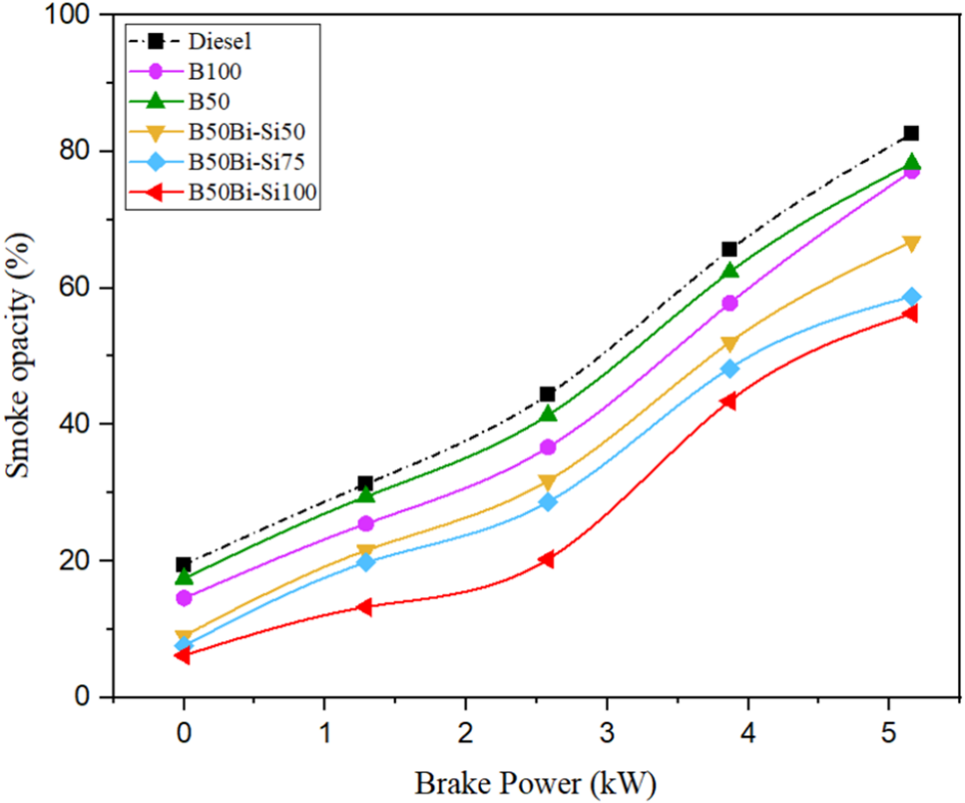
Variations of smoke opacity.

Fuel atomization, oxygen deprivation, inadequate combustion, and fuel self-ignition temperature affect smoke emissions^40^. Figure 13 displays a decrease in the smoke opacity for test blends compared to diesel. For all test fuels, increasing braking power increases smoke opacity. According to Chen et al. [46], there is a positive correlation between brake power and the quantity of fuel supplied per unit of air. This relationship leads to suboptimal oxidation and elevated levels of smoke opacity. At maximum load, the B50Bio-Si100 fuel exhibited a smoke opacity that was 31.87% lower than diesel. This decline was due to oxygen substance in Bio-Si nanoparticles, which urged the combustion of test fuels. Whereas the smoke opacity of each mixture increases by increasing brake power, it reduces as additional Bio-Si is added to the blends. Using nanoparticles has improved ignition properties, shorter delay, and high evaporation rate [47].

#### Hydrocarbon emissions

**Figure 14:**
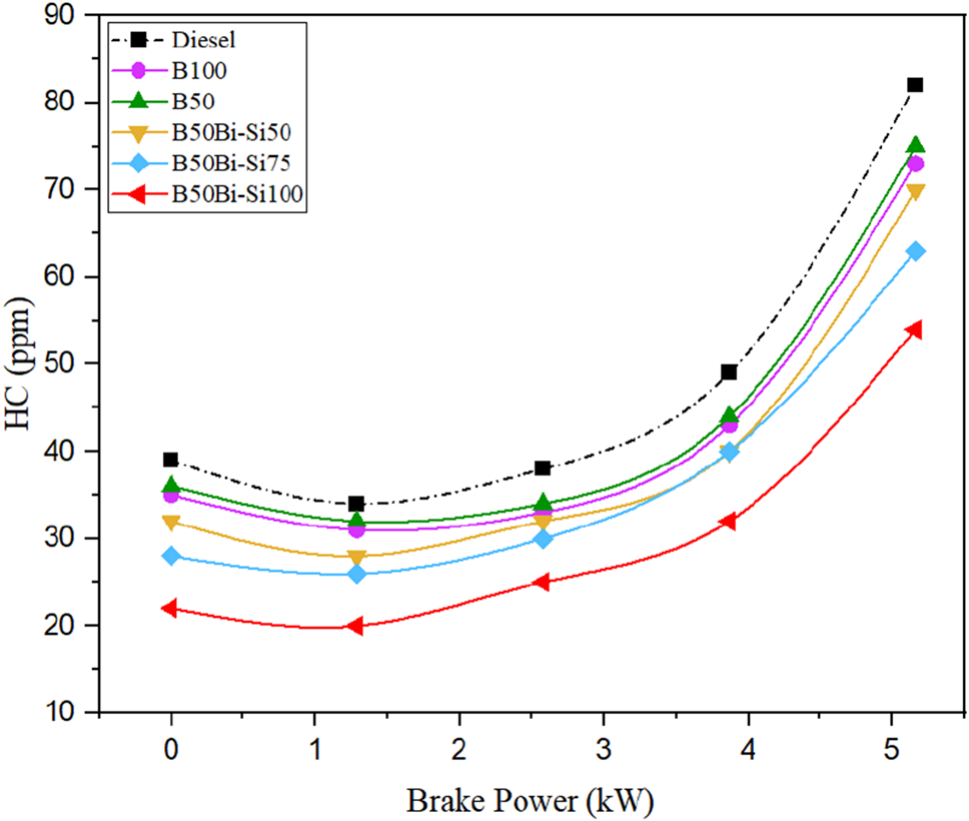
Variations of hydrocarbon emissions.

HC emissions are produced by incomplete combustion [48, 49]. The differences in HC emissions for the test fuels are shown in Fig. 14. As engine brake power increased due to the rich mixes progress, HC emissions increased in all blends [50].

At higher brake power, the HC emissions in the B50Bio-Si100 blend were 34.14% lower than in diesel fuel. Under all conditions, Bio-Si nanoparticle mixes decreased HC emissions more than diesel fuel [46]. Doping of Bio-Si in the biodiesel mixture, fuel combustion is advanced owing to the Bio-Si acting as a catalyst for improving flame propagation and reducing carbon activation temperature. Moreover, the biodiesel blend’s higher oxygen level and cetane number significantly decreased HC emissions [47].

#### Carbon monoxide emission

**Figure 15:**
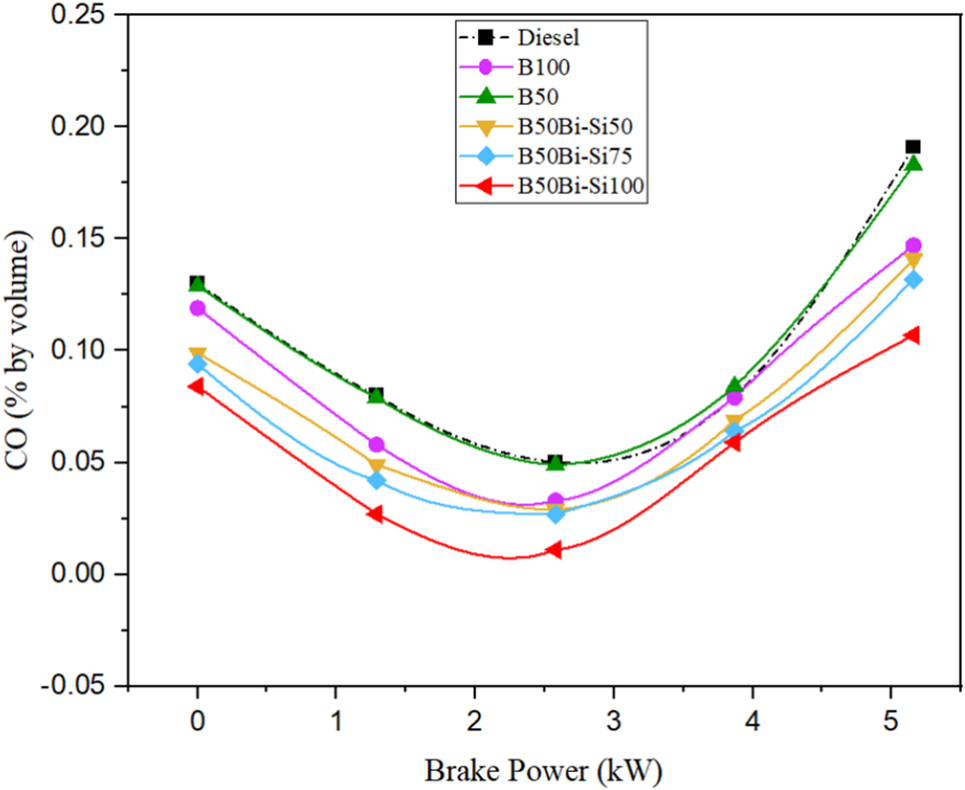
Variations of CO emissions.

The primary factors contributing to CO emissions are inadequate air supply during the combustion process, suboptimal air impingement, deficiencies in fuel mix formation, and partial combustion [51, 52]. Figure 15 depicts the differences in CO emissions among the various test fuels.

Owing to the better oxygen substance in biodiesel and the catalytic properties of Bio-Si, the B50Bio-Si100 blend reduced CO emissions by 43.97% more than diesel fuel^38^. The combustion was discovered to be partial when Bio-Si was eliminated instead of a fuel blend with the right proportion of fuel to air. It reduced CO emissions and produced high-quality complete combustion^27^.

## Conclusion

The biodiesel obtained from leather industry waste fat made through the transesterification method to power CRDI diesel engines is detailed in this analysis. The following are the study’s most important conclusions:

- The peak in-cylinder pressure of B100 was perceived to be 5.80% higher than diesel.
- The blend of B50Bio-Si100 has a higher HRR than other blends but is 5.1% less than diesel.
- The blend B50Bio-Si100 improved BTE by 11.24% more than pure biodiesel at maximum brake power but 2.27% lower than neat diesel fuel.
- Smoke opacity is decreased in all the Bio-Si blends than in diesel fuel. The B50Bio-Si100 blend produced 31.87% lower smoke opacity for diesel fuel at maximum brake power.
- HC emissions were reduced by 34.14%, and CO emissions were reduced by 43.97% in the B50Bio-Si100 blend than diesel fuel at maximum brake power.
- NOx emissions are higher in all the Bio-Si nanoparticle blends than in diesel fuel. The B50Bio-Si100 blend produces 4.35% higher NOx emissions than diesel fuel at maximum brake power.

Based on the results obtained, it can be concluded that the use of LIWFB blends with Bio-Si100 ppm by volume demonstrates effective performance in the context of CRDI diesel engine applications, resulting in reduced emissions overall but with a slight increase in NOx emissions.

## Scope of the future work

Modifying engine operational constraints like compression ratios, injection pressure strategy, injection timing strategy, and exhaust gas recirculation would be utilized to determine the LIWFB in a CRDI diesel engine. To further alter the resulting fuels chemically, oxygenated additives should be added.

## Data Availability

The datasets used and/or analysed during the current study available from the corresponding author on reasonable request.

## Abbreviations

Bio-Si: Bio-silica
BSEC: Brake-specific energy consumption
BTDC: Before top dead centre
CTAB: Cetyl trimethyl ammonium bromide
CuO: Copper oxide
HRR: Heat release rate
HC: Hydrocarbon
LIWFB: Leather industry waste fat biodiesel
